# Ethyl [1-(4-bromo­phen­yl)-1-hydr­oxy-3-oxobut­yl](phen­yl)phosphinate monohydrate^1^

**DOI:** 10.1107/S160053681000437X

**Published:** 2010-02-10

**Authors:** Sampak Samanta, Sandun Perera, Grant A. Broker, Cong-Gui Zhao, Edward R. T. Tiekink

**Affiliations:** a Department of Chemistry, University of Texas at San Antonio, One UTSA Circle, San Antonio, Texas 78249-0698, USA; b Department of Chemistry, University of Malaya, 50603 Kuala Lumpur, Malaysia

## Abstract

In the title hydrate, C_18_H_20_BrO_4_P·H_2_O, a staggered conformation is found when the organic mol­ecule is viewed down the central P—C bond, with the oxo and hydroxyl groups being diagonally opposite; each of the central P and C atoms has an *S*-configuration. The crystal structure features supra­molecular double chains along the *b* axis mediated by O_hydrox­yl_–H⋯O_oxo_, O_water_–H⋯O_oxo_, and O_water_–H⋯O_water_ hydrogen bonds.

## Related literature

For background to the enanti­oselective synthesis of the biologically significant *R*-hydroxy­phosphinates, see: Samanta *et al.* (2010 ▶).
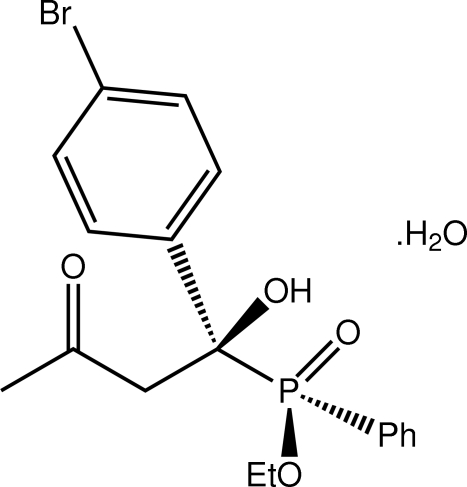


## Experimental

### 

#### Crystal data

C_18_H_20_BrO_4_P·H_2_O
*M* *_r_* = 429.24
Monoclinic, 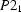

*a* = 10.140 (2) Å
*b* = 5.7779 (12) Å
*c* = 16.691 (3) Å
β = 104.79 (3)°
*V* = 945.5 (3) Å^3^
*Z* = 2
Mo *K*α radiation
μ = 2.28 mm^−1^
*T* = 173 K
0.30 × 0.11 × 0.08 mm

#### Data collection

Rigaku AFC12/SATURN724 diffractometer
Absorption correction: multi-scan (*ABSCOR*; Higashi, 1995 ▶) *T*_min_ = 0.607, *T*_max_ = 1
6979 measured reflections
3568 independent reflections
3382 reflections with *I* > 2σ(*I*)
*R*_int_ = 0.042

#### Refinement

*R*[*F* ^2^ > 2σ(*F* ^2^)] = 0.044
*wR*(*F* ^2^) = 0.094
*S* = 1.06
3568 reflections
236 parameters
5 restraints
H-atom parameters constrained
Δρ_max_ = 0.31 e Å^−3^
Δρ_min_ = −0.37 e Å^−3^
Absolute structure: Flack (1983 ▶), 1408 Friedel pairs
Flack parameter: 0.015 (10)

### 

Data collection: *CrystalClear* (Rigaku/MSC, 2005 ▶); cell refinement: *CrystalClear*; data reduction: *CrystalClear*; program(s) used to solve structure: *SHELXS97* (Sheldrick, 2008 ▶); program(s) used to refine structure: *SHELXL97* (Sheldrick, 2008 ▶); molecular graphics: *ORTEPII* (Johnson, 1976 ▶) and *DIAMOND* (Brandenburg, 2006 ▶); software used to prepare material for publication: *publCIF* (Westrip, 2010 ▶).

## Supplementary Material

Crystal structure: contains datablocks global, I. DOI: 10.1107/S160053681000437X/hb5328sup1.cif

Structure factors: contains datablocks I. DOI: 10.1107/S160053681000437X/hb5328Isup2.hkl

Additional supplementary materials:  crystallographic information; 3D view; checkCIF report

## Figures and Tables

**Table 1:** Hydrogen-bond geometry (Å, °)

*D*—H⋯*A*	*D*—H	H⋯*A*	*D*⋯*A*	*D*—H⋯*A*
O1—H1o⋯O2^i^	0.84	1.94	2.698 (4)	150
O1w—H1w⋯O2	0.84	2.03	2.855 (4)	168
O1w—H2w⋯O1w^ii^	0.84	2.24	3.072 (6)	172

Symmetry codes: (i) 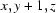; (ii) 
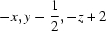
.

**Fig. 1.**
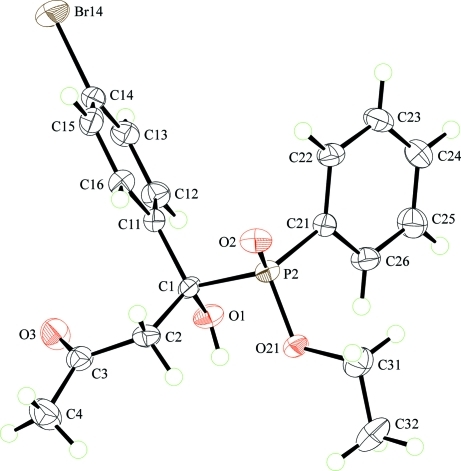
Molecular structure of the molecule in (I), showing displacement ellipsoids at the 50% probability level. The water molecule is not shown.

**Fig. 2.**
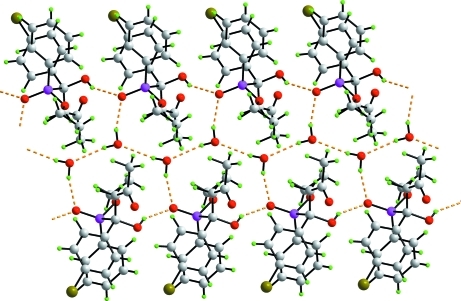
Supramolecular double chain along the b axis in (I) mediated by O–H···O hydrogen bonding (orange dashed lines). Colour scheme: Br, olive; P. pink; O, red; N, blue; C, grey; and H, green.

**Fig. 3.**
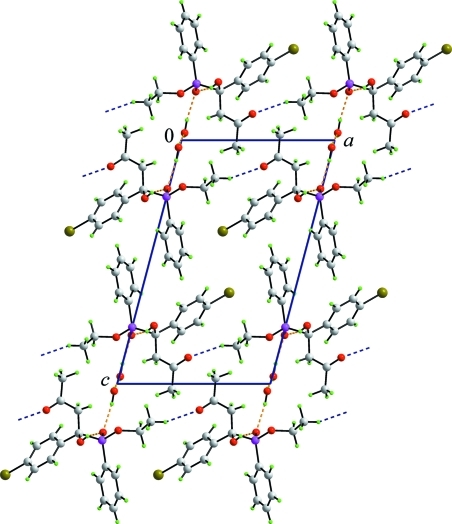
View in projection down the b axis of the unit cell contents in (I) highlighting the formation of layers in the ab plane. The O–H···O hydrogen bonding and C–H···O contacts are shown as orange and blue dashed lines, respectively. Colour scheme: Br, olive; P. pink; O, red; N, blue; C, grey; and H, green.

## supplementary crystallographic information

### Comment

The title hydrate, (I), was investigated as a part of on-going studies on the enantioselective synthesis of the biologically significant *R*-hydroxyphosphinates (Samanta *et al.*, 2010). The crystal structure analysis of diastereoisomer in (I), Fig. 1, confirms the stereochemistry of the C1 atom to be *S* and that of the P2 atom to be likewise *S*. There are significant hydrogen bonding interactions in the structure and the solvent water molecules play a pivotal role in the supramolecular aggregation. Direct links between molecules are found through the agency of O1_hydroxyl_—H1o···O2_oxo_ hydrogen bonds, Fig. 2 and Table 1. Chains thus formed along the *b* direction are linked into double chains via a central zig-zag arrangement of hydrogen bonded water molecules that are connected to phosphorous-oxide atoms, Fig. 2 and Table 1. The combination of these O—H···O interactions leads to the formation of 13-membered {···O═ PCOH···O_oxo_···HO_water_H···O_water_H···O_water_H} synthons. These chains are connected into layers in the *ab* plane via C–H···O interactions, Table 1, involving a methyl-H and carbonyl-O atoms, Fig. 3.

### Experimental

The title compound was prepared as described in the literature (Samanta *et al.*, 2010). Crystals were obtained by dissolving the diastereomeric products obtained in the cross aldol reaction in a minimum amount of ethyl acetate and then diluting with hexane. The solution was kept in the open to allow solvent slow evaporation, and colourless blocks of (I) were obtained after 24 h.

### Refinement

The C-bound H atoms were geometrically placed (C—H = 0.95-0.99 Å) and refined as riding with *U*_iso_(H) = 1.2-1.5*U*_eq_(C). The methyl H-atoms were rotated to fit the electron density. The O–H H atoms were located from a difference map and refined with O–H = 0.840±0.001 Å, and with *U*_iso_(H) = 1.5*U*_eq_(O).

### Crystal data

**Table:**

C_18_H_20_BrO_4_P·H_2_O	*F*(000) = 440
*M**_r_* = 429.24	*D*_x_ = 1.508 Mg m^−^^3^
Monoclinic, *P*2_1_	Mo *K*α radiation, λ = 0.71070 Å
Hall symbol: P 2yb	Cell parameters from 2824 reflections
*a* = 10.140 (2) Å	θ = 2.5–30.2°
*b* = 5.7779 (12) Å	µ = 2.28 mm^−^^1^
*c* = 16.691 (3) Å	*T* = 173 K
β = 104.79 (3)°	Block, colourless
*V* = 945.5 (3) Å^3^	0.30 × 0.11 × 0.08 mm
*Z* = 2	

### Data collection

**Table:**

Rigaku AFC12K/SATURN724 diffractometer	3568 independent reflections
Radiation source: fine-focus sealed tube	3382 reflections with *I* > 2σ(*I*)
graphite	*R*_int_ = 0.042
ω scans	θ_max_ = 26.5°, θ_min_ = 2.5°
Absorption correction: multi-scan (*ABSCOR*; Higashi, 1995)	*h* = −12→12
*T*_min_ = 0.607, *T*_max_ = 1	*k* = −7→6
6979 measured reflections	*l* = −20→17

### Refinement

**Table:**

Refinement on *F*^2^	Secondary atom site location: difference Fourier map
Least-squares matrix: full	Hydrogen site location: inferred from neighbouring sites
*R*[*F*^2^ > 2σ(*F*^2^)] = 0.044	H-atom parameters constrained
*wR*(*F*^2^) = 0.094	*w* = 1/[σ^2^(*F*_o_^2^) + (0.0353*P*)^2^ + 0.386*P*] where *P* = (*F*_o_^2^ + 2*F*_c_^2^)/3
*S* = 1.06	(Δ/σ)_max_ = 0.001
3568 reflections	Δρ_max_ = 0.31 e Å^−^^3^
236 parameters	Δρ_min_ = −0.37 e Å^−^^3^
5 restraints	Absolute structure: Flack (1983), 1408 Friedel pairs
Primary atom site location: structure-invariant direct methods	Flack parameter: 0.015 (10)

### Special details

**Table:**

Geometry. All esds (except the esd in the dihedral angle between two l.s. planes) are estimated using the full covariance matrix. The cell esds are taken into account individually in the estimation of esds in distances, angles and torsion angles; correlations between esds in cell parameters are only used when they are defined by crystal symmetry. An approximate (isotropic) treatment of cell esds is used for estimating esds involving l.s. planes.
Refinement. Refinement of *F*^2^ against ALL reflections. The weighted *R*-factor wR and goodness of fit *S* are based on *F*^2^, conventional *R*-factors *R* are based on *F*, with *F* set to zero for negative *F*^2^. The threshold expression of *F*^2^ > σ(*F*^2^) is used only for calculating *R*-factors(gt) etc. and is not relevant to the choice of reflections for refinement. *R*-factors based on *F*^2^ are statistically about twice as large as those based on *F*, and *R*- factors based on ALL data will be even larger.

### Fractional atomic coordinates and isotropic or equivalent isotropic displacement parameters (Å^2^)

**Table:**

	*x*	*y*	*z*	*U*_iso_*/*U*_eq_	
Br14	0.57412 (4)	−0.25929 (15)	0.62623 (3)	0.04821 (16)	
P2	0.00084 (10)	0.0452 (2)	0.76819 (6)	0.0228 (2)	
O1	0.1397 (3)	0.4301 (5)	0.76143 (18)	0.0293 (6)	
H1O	0.0981	0.5136	0.7882	0.044*	
O1W	0.0072 (4)	−0.2519 (7)	0.97010 (18)	0.0583 (9)	
H1W	0.0023	−0.2549	0.9191	0.087*	
H2W	0.0093	−0.3917	0.9841	0.087*	
O2	0.0088 (3)	−0.1965 (4)	0.80037 (15)	0.0291 (6)	
O3	0.4175 (3)	0.4060 (6)	0.89220 (18)	0.0380 (7)	
O21	−0.0871 (2)	0.2144 (5)	0.80840 (14)	0.0259 (5)	
C1	0.1642 (3)	0.2055 (7)	0.7962 (2)	0.0220 (8)	
C2	0.2089 (4)	0.2109 (7)	0.8912 (2)	0.0276 (8)	
H2A	0.1347	0.2829	0.9113	0.033*	
H2B	0.2182	0.0491	0.9114	0.033*	
C3	0.3397 (4)	0.3364 (6)	0.9309 (2)	0.0283 (9)	
C4	0.3666 (5)	0.3686 (9)	1.0232 (3)	0.0445 (12)	
H4A	0.3941	0.2205	1.0511	0.067*	
H4B	0.2835	0.4240	1.0367	0.067*	
H4C	0.4398	0.4822	1.0419	0.067*	
C11	0.2671 (4)	0.0904 (6)	0.7559 (2)	0.0228 (8)	
C12	0.3013 (4)	0.1955 (7)	0.6892 (2)	0.0309 (9)	
H12	0.2608	0.3397	0.6693	0.037*	
C13	0.3937 (4)	0.0940 (7)	0.6509 (2)	0.0296 (9)	
H13	0.4178	0.1684	0.6059	0.035*	
C14	0.4496 (4)	−0.1171 (7)	0.6798 (2)	0.0297 (9)	
C15	0.4176 (4)	−0.2265 (7)	0.7454 (2)	0.0299 (9)	
H15	0.4573	−0.3718	0.7644	0.036*	
C16	0.3267 (4)	−0.1221 (7)	0.7834 (2)	0.0262 (8)	
H16	0.3046	−0.1966	0.8290	0.031*	
C21	−0.0698 (4)	0.0655 (7)	0.6594 (2)	0.0249 (8)	
C22	−0.0543 (4)	−0.1151 (7)	0.6068 (2)	0.0303 (9)	
H22	−0.0024	−0.2481	0.6286	0.036*	
C23	−0.1158 (4)	−0.0985 (8)	0.5218 (3)	0.0336 (10)	
H23	−0.1061	−0.2214	0.4859	0.040*	
C24	−0.1898 (4)	0.0934 (7)	0.4901 (3)	0.0367 (10)	
H24	−0.2318	0.1019	0.4323	0.044*	
C25	−0.2041 (4)	0.2751 (8)	0.5410 (2)	0.0389 (10)	
H25	−0.2547	0.4085	0.5182	0.047*	
C26	−0.1447 (4)	0.2630 (8)	0.6256 (2)	0.0302 (8)	
H26	−0.1547	0.3878	0.6606	0.036*	
C31	−0.2290 (4)	0.1602 (8)	0.8061 (3)	0.0384 (10)	
H31A	−0.2337	0.0163	0.8375	0.046*	
H31B	−0.2827	0.1376	0.7482	0.046*	
C32	−0.2844 (5)	0.3569 (9)	0.8440 (3)	0.0504 (13)	
H32A	−0.3797	0.3257	0.8431	0.076*	
H32B	−0.2792	0.4983	0.8125	0.076*	
H32C	−0.2309	0.3771	0.9014	0.076*	

### Atomic displacement parameters (Å^2^)

**Table:**

	*U*^11^	*U*^22^	*U*^33^	*U*^12^	*U*^13^	*U*^23^
Br14	0.0388 (2)	0.0579 (3)	0.0544 (3)	0.0052 (2)	0.0236 (2)	−0.0160 (2)
P2	0.0238 (5)	0.0223 (5)	0.0239 (5)	0.0017 (4)	0.0092 (4)	−0.0009 (4)
O1	0.0350 (16)	0.0203 (13)	0.0357 (16)	0.0046 (12)	0.0149 (13)	0.0010 (11)
O1W	0.086 (2)	0.058 (2)	0.0360 (17)	−0.006 (3)	0.0257 (18)	−0.0023 (19)
O2	0.0414 (16)	0.0215 (14)	0.0278 (13)	0.0016 (11)	0.0150 (12)	0.0025 (10)
O3	0.0343 (17)	0.0476 (18)	0.0342 (16)	−0.0089 (14)	0.0123 (14)	−0.0023 (14)
O21	0.0224 (12)	0.0297 (14)	0.0284 (12)	0.0014 (12)	0.0116 (10)	−0.0040 (12)
C1	0.0196 (17)	0.024 (2)	0.0237 (17)	0.0055 (15)	0.0068 (14)	−0.0003 (15)
C2	0.0313 (19)	0.030 (2)	0.0242 (18)	0.0019 (18)	0.0116 (15)	−0.0036 (17)
C3	0.031 (2)	0.0249 (19)	0.029 (2)	0.0062 (16)	0.0062 (17)	0.0001 (16)
C4	0.045 (3)	0.058 (3)	0.030 (2)	−0.009 (2)	0.010 (2)	−0.009 (2)
C11	0.0190 (18)	0.0242 (19)	0.0243 (18)	0.0002 (14)	0.0040 (15)	−0.0031 (15)
C12	0.036 (2)	0.033 (2)	0.0255 (19)	0.0036 (17)	0.0092 (17)	0.0021 (17)
C13	0.034 (2)	0.033 (2)	0.0239 (19)	−0.0012 (17)	0.0110 (17)	−0.0008 (17)
C14	0.023 (2)	0.035 (2)	0.032 (2)	−0.0029 (17)	0.0074 (17)	−0.0130 (18)
C15	0.0232 (19)	0.028 (2)	0.037 (2)	0.0017 (17)	0.0047 (16)	−0.0001 (18)
C16	0.026 (2)	0.0239 (19)	0.0293 (19)	0.0006 (16)	0.0093 (16)	0.0020 (16)
C21	0.0227 (19)	0.0254 (19)	0.0264 (19)	−0.0001 (15)	0.0059 (15)	−0.0036 (15)
C22	0.033 (2)	0.026 (2)	0.031 (2)	0.0067 (17)	0.0069 (18)	−0.0040 (17)
C23	0.039 (2)	0.033 (2)	0.028 (2)	−0.0032 (19)	0.0086 (19)	−0.0100 (17)
C24	0.037 (2)	0.044 (3)	0.027 (2)	0.000 (2)	0.0024 (18)	−0.0053 (19)
C25	0.043 (2)	0.039 (3)	0.031 (2)	0.007 (2)	0.0025 (18)	0.002 (2)
C26	0.036 (2)	0.027 (2)	0.0284 (18)	0.0062 (19)	0.0099 (16)	−0.0020 (18)
C31	0.031 (2)	0.045 (3)	0.044 (2)	−0.0021 (18)	0.018 (2)	−0.0023 (19)
C32	0.037 (3)	0.057 (3)	0.064 (3)	0.017 (2)	0.024 (2)	0.004 (3)

### Geometric parameters (Å, °)

**Table:**

Br14—C14	1.909 (4)	C13—C14	1.379 (6)
P2—O2	1.491 (2)	C13—H13	0.9500
P2—O21	1.584 (3)	C14—C15	1.373 (5)
P2—C21	1.778 (4)	C15—C16	1.383 (5)
P2—C1	1.850 (4)	C15—H15	0.9500
O1—C1	1.418 (5)	C16—H16	0.9500
O1—H1O	0.8400	C21—C22	1.397 (5)
O1W—H1W	0.8400	C21—C26	1.407 (5)
O1W—H2W	0.8400	C22—C23	1.400 (6)
O3—C3	1.209 (5)	C22—H22	0.9500
O21—C31	1.464 (5)	C23—C24	1.368 (6)
C1—C11	1.531 (5)	C23—H23	0.9500
C1—C2	1.534 (4)	C24—C25	1.382 (6)
C2—C3	1.508 (5)	C24—H24	0.9500
C2—H2A	0.9900	C25—C26	1.388 (5)
C2—H2B	0.9900	C25—H25	0.9500
C3—C4	1.506 (5)	C26—H26	0.9500
C4—H4A	0.9800	C31—C32	1.479 (6)
C4—H4B	0.9800	C31—H31A	0.9900
C4—H4C	0.9800	C31—H31B	0.9900
C11—C12	1.388 (5)	C32—H32A	0.9800
C11—C16	1.393 (5)	C32—H32B	0.9800
C12—C13	1.392 (5)	C32—H32C	0.9800
C12—H12	0.9500		
			
O2—P2—O21	114.20 (15)	C13—C14—C15	121.8 (4)
O2—P2—C21	113.59 (17)	C13—C14—Br14	118.6 (3)
O21—P2—C21	105.60 (16)	C15—C14—Br14	119.6 (3)
O2—P2—C1	114.53 (16)	C14—C15—C16	119.0 (4)
O21—P2—C1	98.54 (15)	C14—C15—H15	120.5
C21—P2—C1	109.05 (17)	C16—C15—H15	120.5
C1—O1—H1O	111.5	C15—C16—C11	121.2 (4)
H1W—O1W—H2W	104.7	C15—C16—H16	119.4
C31—O21—P2	120.9 (2)	C11—C16—H16	119.4
O1—C1—C11	106.6 (3)	C22—C21—C26	119.3 (3)
O1—C1—C2	112.3 (3)	C22—C21—P2	120.8 (3)
C11—C1—C2	114.1 (3)	C26—C21—P2	119.9 (3)
O1—C1—P2	107.6 (2)	C21—C22—C23	119.5 (4)
C11—C1—P2	109.7 (2)	C21—C22—H22	120.2
C2—C1—P2	106.3 (2)	C23—C22—H22	120.2
C3—C2—C1	117.3 (3)	C24—C23—C22	120.4 (4)
C3—C2—H2A	108.0	C24—C23—H23	119.8
C1—C2—H2A	108.0	C22—C23—H23	119.8
C3—C2—H2B	108.0	C23—C24—C25	120.7 (4)
C1—C2—H2B	108.0	C23—C24—H24	119.6
H2A—C2—H2B	107.2	C25—C24—H24	119.6
O3—C3—C2	123.1 (3)	C24—C25—C26	120.1 (4)
O3—C3—C4	122.2 (4)	C24—C25—H25	120.0
C2—C3—C4	114.7 (4)	C26—C25—H25	120.0
C3—C4—H4A	109.5	C25—C26—C21	119.9 (4)
C3—C4—H4B	109.5	C25—C26—H26	120.1
H4A—C4—H4B	109.5	C21—C26—H26	120.1
C3—C4—H4C	109.5	O21—C31—C32	107.6 (4)
H4A—C4—H4C	109.5	O21—C31—H31A	110.2
H4B—C4—H4C	109.5	C32—C31—H31A	110.2
C12—C11—C16	118.3 (3)	O21—C31—H31B	110.2
C12—C11—C1	120.0 (3)	C32—C31—H31B	110.2
C16—C11—C1	121.7 (3)	H31A—C31—H31B	108.5
C11—C12—C13	121.3 (4)	C31—C32—H32A	109.5
C11—C12—H12	119.4	C31—C32—H32B	109.5
C13—C12—H12	119.4	H32A—C32—H32B	109.5
C14—C13—C12	118.5 (4)	C31—C32—H32C	109.5
C14—C13—H13	120.8	H32A—C32—H32C	109.5
C12—C13—H13	120.8	H32B—C32—H32C	109.5
			
O2—P2—O21—C31	−55.9 (3)	C1—C11—C12—C13	−179.5 (3)
C21—P2—O21—C31	69.7 (3)	C11—C12—C13—C14	1.1 (6)
C1—P2—O21—C31	−177.7 (3)	C12—C13—C14—C15	−0.8 (5)
O2—P2—C1—O1	−179.9 (2)	C12—C13—C14—Br14	179.0 (3)
O21—P2—C1—O1	−58.3 (2)	C13—C14—C15—C16	0.1 (6)
C21—P2—C1—O1	51.5 (3)	Br14—C14—C15—C16	−179.7 (3)
O2—P2—C1—C11	64.5 (3)	C14—C15—C16—C11	0.4 (6)
O21—P2—C1—C11	−173.9 (2)	C12—C11—C16—C15	−0.1 (5)
C21—P2—C1—C11	−64.1 (3)	C1—C11—C16—C15	178.8 (3)
O2—P2—C1—C2	−59.4 (3)	O2—P2—C21—C22	−29.9 (4)
O21—P2—C1—C2	62.2 (3)	O21—P2—C21—C22	−155.8 (3)
C21—P2—C1—C2	172.1 (2)	C1—P2—C21—C22	99.1 (3)
O1—C1—C2—C3	−61.6 (4)	O2—P2—C21—C26	148.7 (3)
C11—C1—C2—C3	59.9 (5)	O21—P2—C21—C26	22.8 (4)
P2—C1—C2—C3	−179.0 (3)	C1—P2—C21—C26	−82.3 (3)
C1—C2—C3—O3	−9.4 (6)	C26—C21—C22—C23	−1.3 (6)
C1—C2—C3—C4	170.7 (4)	P2—C21—C22—C23	177.3 (3)
O1—C1—C11—C12	−9.3 (4)	C21—C22—C23—C24	0.5 (6)
C2—C1—C11—C12	−133.8 (4)	C22—C23—C24—C25	0.6 (7)
P2—C1—C11—C12	107.0 (3)	C23—C24—C25—C26	−0.9 (7)
O1—C1—C11—C16	171.9 (3)	C24—C25—C26—C21	0.1 (6)
C2—C1—C11—C16	47.4 (5)	C22—C21—C26—C25	1.0 (6)
P2—C1—C11—C16	−71.8 (4)	P2—C21—C26—C25	−177.6 (3)
C16—C11—C12—C13	−0.7 (5)	P2—O21—C31—C32	−176.7 (3)

### Hydrogen-bond geometry (Å, °)

**Table:**

*D*—H···*A*	*D*—H	H···*A*	*D*···*A*	*D*—H···*A*
O1—H1o···O2^i^	0.84	1.94	2.698 (4)	150
O1w—H1w···O2	0.84	2.03	2.855 (4)	168
O1w—H2w···O1w^ii^	0.84	2.24	3.072 (6)	172

Symmetry codes: (i) *x*, *y*+1, *z*;  (ii) −*x*, *y*−1/2, −*z*+2.
